# Safety Related to the Timing of Radiotherapy and Immune Checkpoint Inhibitors in Patients with Advanced Non-Small Cell Lung Cancer: A Single Institutional Experience

**DOI:** 10.3390/curroncol29010021

**Published:** 2022-01-07

**Authors:** Michael C. Tjong, Malavan Ragulojan, Ian Poon, Alexander V. Louie, Susanna Y. Cheng, Mark Doherty, Liying Zhang, Yee Ung, Patrick Cheung, Parneet K. Cheema

**Affiliations:** 1 Department of Radiation Oncology, Sunnybrook Odette Cancer Centre, Sunnybrook Hospital, Toronto, ON M4N3M5, Canada; michael.tjong@rmp.uhn.ca (M.C.T.); alexander.louie@sunnybrook.ca (A.V.L.); zhangliege@gmail.com (L.Z.); yee.ung@sunnybrook.ca (Y.U.); patrick.cheung@sunnybrook.ca (P.C.); 2 Faculty of Medicine, McMaster University, Hamilton, ON L8S4L8, Canada; malavan.ragulojan@medportal.ca; 3 Department of Medical Oncology and Hematology, Sunnybrook Odette Cancer Centre, Sunnybrook Hospital, Toronto, ON M4N3M5, Canada; susanna.cheng@sunnybrook.ca (S.Y.C.); markdoherty@svhg.ie (M.D.); 4 Department of Medical Oncology and Hematology, William Osler Health System, Brampton, ON L6R3J7, Canada

**Keywords:** radiotherapy, immunotherapy, timing, safety, toxicity

## Abstract

Background: The safety impact of radiotherapy (RT) timing relative to immune checkpoint inhibitors (ICIs) for advanced non-small-cell lung cancer (NSCLC) is unclear. We investigated if RT within 14 days (Interval 1) and 90 days (Interval 2) of ICI use is associated with toxicities compared to RT outside these intervals. Methods: Advanced NSCLC patients treated with both RT and ICIs were reviewed. Toxicities were graded as per CTCAE v4.0 and attributed to either ICIs or RT by clinicians. Associations between RT timing and Grade ≥2 toxicities were analyzed using logistic regression models adjusted for patient, disease, and treatment factors (α = 0.05). Results: Sixty-four patients were identified. Twenty received RT within Interval 1 and 40 within Interval 2. There were 20 Grade ≥2 toxicities in 18 (28%) patients; pneumonitis (6) and nausea (2) were most prevalent. One treatment-related death (immune encephalitis) was observed. Rates of patients with Grade ≥2 toxicities were 35%/25% in the group with/without RT within Interval 1 and 30%/25% in the group with/without RT within Interval 2. No significant association between RT timing relative to ICI use period and Grade ≥2 toxicities was observed. Conclusion: Albeit limited by the small sample size, the result suggested that pausing ICIs around RT use may not be necessary.

## 1. Introduction

Metastatic non-small cell lung cancer (NSCLC) is associated with poor outcome, with an associated 5-year survival of 7% according to Surveillance, Epidemiology, and End Results (SEER) data from 2008 to 2014 [1]. Recent advances have established immune checkpoint inhibitors (ICIs), including PD-1/PD-L1 inhibitors as standard-of-care treatment options for locally advanced and metastatic NSCLC patients [2,3,4,5,6,7,8,9]. These ICIs enhance immune-mediated anticancer activity by blocking immune-attenuating interaction between the PD-1 receptor on T-lymphocytes and PD-L1 on cancer cells [10]. In multiple randomized trials, the use of ICIs has been associated with improved overall survival (OS) compared to cytotoxic chemotherapy in the setting of de novo and previously treated metastatic NSCLC [5,6,7,8,9]. Although ICIs are generally well tolerated, they can be associated with immune-related toxicities such as pneumonitis, colitis, and dermatitis [2,8].

Alongside systemic therapies, many patients with advanced NSCLC patients may benefit from radiotherapy (RT) in conventional or ablative fractionations to provide local control or symptom relief [11]. Information related to the safety of RT in patients receiving palliative-intent ICIs based on timing and specific dose and fractionations is evolving. As side effects from RT can include inflammatory toxicities such as radiation pneumonitis and enteritis, the addition of RT to ICIs may synergistically lead to increased severity and incidence of immune-mediated toxicities [12]. RT is often delivered in close timing with ICI treatment for a variety of reasons, including urgent symptom control or oligoprogression while minimizing delays to ICI dosing. Moreover, the role of RT was not adequately described in many major randomized trials investigating ICIs for advanced NSCLC patients raising the concern of the use of RT with ICIs unrecognized deleterious effects [6,7,8]. Secondary analysis of KEYNOTE-001 showed patients who had a history of RT prior to pembrolizumab use had better OS and progression-free survival (PFS) compared to patients who did not, with no significant increase in overall Grade ≥3 pulmonary toxicities [13]. The PEMBRO-RT study evaluating SBRT within 7 days of initiation of ICI reported the rate of ICI-related toxicity as expected with a single-agent ICI of 17% [14]. Nonetheless, whether this safety profile is affected by the length of the period between RT and ICI when both are delivered is not yet well established.

This study specifically investigates the safety impact of radiotherapy timing and dose fractionation schedules among an advanced NSCLC cohort that all received both RT and palliative-intent ICIs. The purpose of this study is to investigate whether RT within 14 days and 90 days of ICI use were associated with increased patients’ odds of Grade ≥2 toxicities as per Common Terminology Criteria for Adverse Events (CTCAE) v4.0 compared to RT outside these intervals. As the half-lives of commonly used PD-1/PD-L1 inhibitors in advanced NSCLC patients range between 3 and 4 weeks, elucidating the safety of RT around the timing of ICI will also help clinical decision making to proceed or delay RT during the period [15].

## 2. Materials and Methods

Patients with advanced NSCLC who received palliative-intent PD-1/PD-L1 inhibitors (pembrolizumab, nivolumab, durvalumab, or atezolizumab) between June 2014 to July 2019 who also had a history of RT (e.g., prior curative-intent and/or palliative-intent regimens) for the NSCLC diagnosis at the tertiary cancer centre were identified and retrospectively reviewed. The study was approved by and adhered to the guidelines and regulations of our institution’s Research Ethics Board (REB). Patient demographic, disease, and clinical information was collected from the institution’s Electronic Patient’s Record. The dates and details related to ICIs and RT delivery were collected and reported. As a wide variety of RT dose regimens were employed in multiple different sites, we converted these to a standardized biologically effective dose (BED_α/β_), which was calculated using the formula: BED_α/β_ $=\mathrm{nd}\left(1+\frac{d}{\alpha /\beta }\right)$, where n is the total number of fractions, d is the dose per fraction, and α/β is the tumor alpha/beta ratio. The value of 10 Gy was used for α/β in calculations. Data on toxicity events after the administration of ICIs were reviewed, collected, and classified by two independent reviewers based on patient chart and imaging information.

### Statistical Analysis

**Figure 1:**
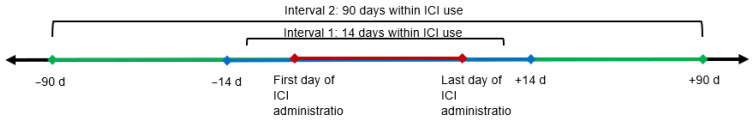
Illustration of RT timing intervals according to immune checkpoint inhibitor (ICI) use period. Controls are patients receiving RT outside of Interval 1 or 2.

Demographics, disease, and treatment characteristics were summarized in total patients using median (interquartiles) for continuous variables and proportions for categorical variables. For each patient, the interval of ICI use was defined as the first and last day of the agent’s administration. Intervals of RT courses from each patient were identified and assessed whether the courses were within 14 or 90 days prior or after the period of ICI use. Patients were grouped based on whether they had any RT within: Interval 1 (14 days prior or after ICI use interval(s)) and Interval 2 (90 days prior or after ICI use interval(s)); (Figure 1). The Wilcoxon rank-sum test for continuous variables and the Fisher’s exact test for categorical variables were performed to compare baseline characteristics between patients within and outside these intervals.

The crude rates of Grade ≥2 toxicities between RT timing groups were reported. Association between RT timing groups with odds of Grade ≥2 toxicities was analyzed using logistic regression UVA and MVA, adjusting for age, histology, ECOG performance status, PD-L1 tumour expression (<1%, 1–49%, or >50%), ICI agents, RT dose regimens (ablative, radical conventional, palliative, or combinations), and RT target site (intracranial vs. non-intracranial), with a value of *p* < 0.05 considered statistically significant. Odds ratio (OR) and 95% CI were also estimated in the logistic regression models. Lastly, OS in the whole cohort and each RT timing group were estimated with Kaplan–Meier methods. All analyses were conducted using Statistical Analysis Software (SAS version 9.4, Cary, NC, USA) and R package (v3.6.1).

## 3. Results

A total of 64 advanced NSCLC patients (63 Stage IV, 1 Stage IIIB) with a median age of 70 years (interquartile range (IQR): 64–77) who received RT and ICIs between June 2014 and July 2019 were identified. One patient with Stage IIIB disease had a cN3 disease and started on pembrolizumab as the disease extent precluded the patient from upfront radical chemoradiation. The median follow-up period from the first day of ICI use was 9.7 (range: 3.1–20.3) months. Forty-nine patients (77%) were alive at the end of the follow-up period. Overall patient baseline and disease characteristics and comparison between patients within and outside Interval 1 or 2 are reported in Table 1. Aside from the lower number of Caucasian patients receiving RT within Interval 2 (68% vs. 96%, *p* = 0.014), there were no significant differences in patient baseline and disease characteristics, including age, ECOG status, stage, histology, and tumour PD-L1, EGFR, and ALK status (Table 1). Atezolizumab and durvalumab were more commonly received by patients receiving RT exclusively outside Interval 2 (*p* = 0.039). Prior to ICI use, 33 patients (52%) received steroid after RT. Interestingly, steroid use after ICI was less frequent among patients receiving RT within Intervals 1 (15% vs. 80%, *p* < 0.001) and 2 (40% vs. 92%, *p* < 0.001).

### 3.1. Immunotherapy and Radiotherapy Details

The median duration of use and number of ICI cycles was 4.2 months and 6.5 cycles, respectively. Immune checkpoint inhibitors used were: nivolumab (45%), pembrolizumab (38%), atezolizumab (9%), and durvalumab (8%). A total of 291 RT courses (143 intracranial, 118 extracranial) were delivered to 64 patients: 97 palliative (dose 8–30 Gy in 1–10 fractions), 173 ablative (16–50 Gy in 1–5 fractions), and 21 conventional radical (dose: 40–78 Gy in 15–39 fractions; mostly as initial treatment in locally advanced setting) doses. Twenty-eight patients received intracranial RT regimens (143 courses total: 122 ablative, 21 palliative): 14 had ablative only, 6 palliative only, and 8 had both ablative and palliative intracranial RT. A total of 20 patients received at least 1 RT course within Interval 1, while 40 patients had RT within Interval 2. Details of radiotherapy regimens between groups based on RT timing relative to ICI are reported in Table 2.

### 3.2. Toxicities

Overall, there were 20 Grade ≥2 toxicity events (2 RT related, 18 ICI related) among 18 (28%) patients (Table 2 and Table 3). Twelve (19%) patients had Grade ≥3 toxicities, and five were pneumonitis (1 RT related, 4 ICI related). One Grade 5 encephalitis (fatality) was reported in a patient who received pembrolizumab 24 days after completing palliative whole-brain radiotherapy (20 Gy in 5 fractions). Two RT-triggered toxicities were: (1) Grade 3 pneumonitis after palliative thoracic RT (30 Gy in 10 fractions) while the patient was on maintenance nivolumab; (2) Grade 2 esophagitis post-palliative cervical spine RT (20 Gy in 5 fractions), followed by Cycle 1 of pembrolizumab a day after RT completion. The full breakdown of Grade ≥2 toxicities is shown in Table 3. Crude rates of patients with Grade ≥2 toxicities were 35%/25% in the group with/without RT within Interval 1 and 30%/25% in the group with/without RT within Interval 2 (Table 2). ICI use was delayed or stopped due to toxicities in four (20%) and nine (23%) patients with RT within Intervals 1 and 2, respectively.

In UVA logistic regression analyses, patient receiving RT within 14 days (OR: 1.62, *p* = 0.41) and 90 days (OR: 1.29, *p* = 0.67) were not significantly associated with Grade ≥2 toxicities (Table 4) compared to patients receiving RT exclusively outside of these intervals. After adjusting for age, histology, ECOG status, PD-L1 status, ICI agents, RT dose regimens (ablative, radical conventional, palliative, or combinations), and RT target site (intracranial vs. non-intracranial) in logistic regression MVA (Table 5), there was no significant association of Grade ≥2 toxicities with RT timing within Interval 1 (OR: 2.34 with 95% CI of 0.47–15.31, *p* = 0.34) or Interval 2 (OR: 1.79 with 95% CI of 0.40–8.93, *p* = 0.48). Neither RT dose regimens nor target sites were associated with toxicities in the final MVA model.

### 3.3. Survival

**Figure 2:**
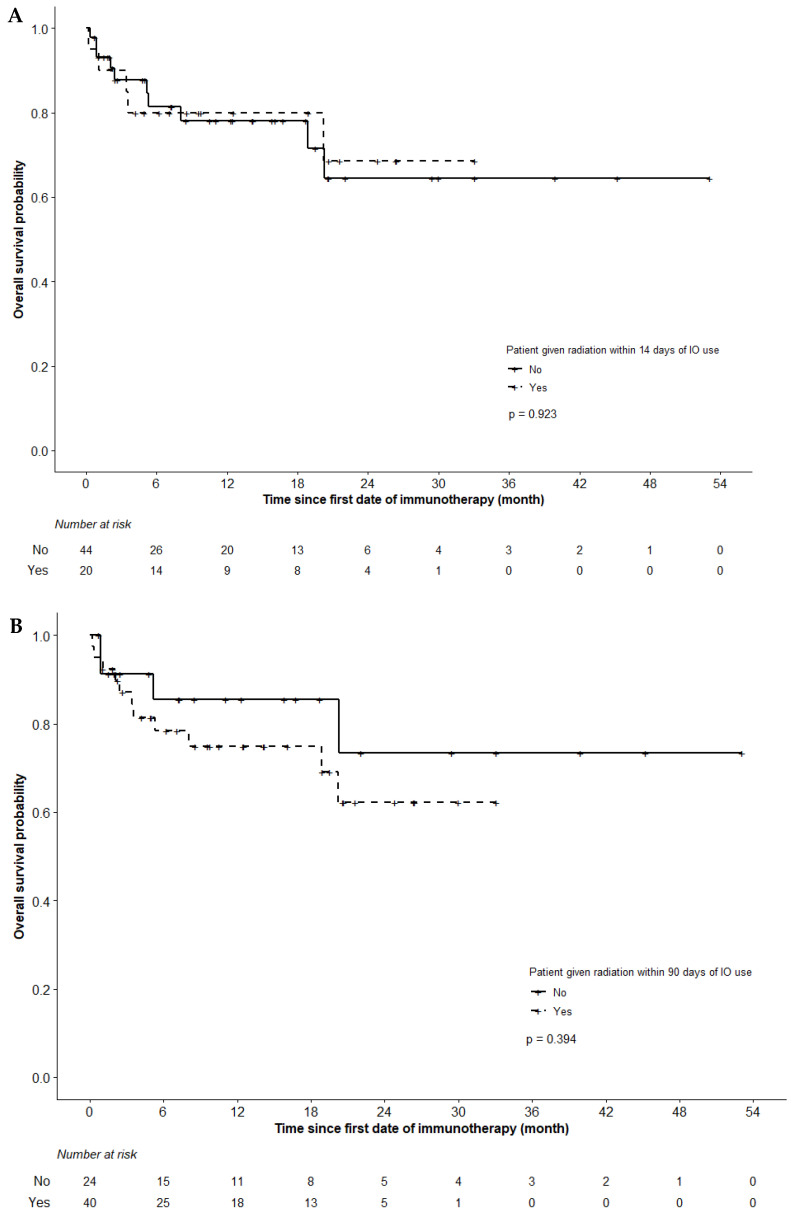
Overall survival based on whether patients had (**A**) RT within 14 days of ICI use (Interval 1) and (**B**) RT within 90 days of ICI use (Interval 2).

In the whole cohort, 1- and 2-year OS was 79% and 66%; actuarial median OS was not reached at the end of the follow-up period. OS at 1 year was 80% among patients who had RT within Interval 1 and 78% among those who only had RT outside of Interval 1 (Figure 2A). Meanwhile, 1-year OS was 75% among patients who only had RT within Interval 2 and 86% among those who had RT outside the interval (Figure 2B). RT in Interval 1 or Interval 2 was not significantly associated with OS (*p* > 0.05).

## 4. Discussion

Our cohort of patients with advanced NSCLC treated with both RT and ICIs demonstrated high 1- and 2-year OS of 79% and 66% after ICI initiation, respectively, albeit with a short median follow-up period of 9.7 months. The results suggested RT within 14 and 90 days of ICI use was not associated with increased mortality compared to RT outside these intervals in patients with advanced NSCLC. The rate of Grade ≥3 toxicities of 17% (ICI related) and 19% (ICI or RT related) in our cohort is similar to rates reported in several prospective trials investigating ICIs for advanced NSCLC (10–27%) [5,6,8] and those with SBRT and ICI (17%) [14]. Pneumonitis (9%) was the most common observed Grade ≥2 adverse event. This indicated that overall, RT is reasonably safe in patients with a history of ICI use and vice versa. With preliminary evidence of increased PFS, OS in patients receiving ICI with a history of RT use in the Phase 1 KEYNOTE-001 of pembrolizumab, concurrent or sequential RT, and ICI use in an advanced NSCLC setting may yield benefit with minimal excess toxicities [13].

Prior studies demonstrated the safety of thoracic RT in patients receiving ICI. Voong et al.’s study with 188 advanced NSCLC patients treated with ICI showed no increase in immune-related pneumonitis in patients with prior thoracic RT [16]. Similarly, in a study with 164 metastatic NSCLC patients treated with ICI, Hwang et al. reported no association between thoracic RT and Grade ≥2 overall adverse events and pneumonitis [17]. A recent randomized trial with 92 advanced NSCLC patients demonstrated no increased toxicity with the addition of stereotactic ablative radiotherapy 7 days prior to pembrolizumab use [14]. Finally, the PACIFIC trial, a large, randomized trial of definitive chemoradiation followed by durvalumab within 6 weeks of radiation, did not seem to increase the rate of Grade 3–4 pneumonitis, and the rate of ICI-related toxicity was similar to previous monotherapy PD1/PDL1 trials. Nonetheless, these studies did not directly investigate the effect of timing between RT and ICI and safety. If a longer period between RT and ICI reduces toxicities, it may be prudent to prolong the interval between two treatments when possible. This is especially important in metastatic, palliative settings, where maintaining immediate comfort and quality of life by minimizing acute toxicity is paramount.

While we could not identify any significant association between RT within Interval 1 or 2 and the odds of developing Grade ≥2 toxicities in UVA and MVA, caution is still needed for RT in these settings. There were nonsignificant trends of increased OR of Grade ≥2 toxicities in patients receiving RT within Interval 1 (1.61) and Interval 2 (1.29); these trends persisted when adjusted to selected clinical factors in MVA with ORs of 2.34 and 1.79 for RT in Intervals 1 and 2, respectively. Clinicians may have been exercising added caution when delivering RT around ICI use, reflected by a lower median BED_10_ of RT courses within Interval 1 (35 Gy_10_ compared to the overall median of 50 Gy_10_). Moreover, perhaps clinicians preselected patients who tolerated ICI well to be receiving RT close to ICI administration. In our cohort, patients receiving RT within these intervals were significantly less likely to be needing steroid after ICI use compared to patients receiving RT exclusively outside these intervals (Table 1).

Nonetheless, with no significant increase in toxicity or deaths, our data suggested that RT within 14 or 90 days of ICI use is likely safe compared to RT outside this interval, albeit with caution needed. Moreover, our rate of severe (Grade ≥ 3) toxicities of 19% in our cohort of advanced NSCLC patients treated with both ICI and RT was within the range of reported rates from published prospective ICI trials where not all patients received RT [5,6,8]. The main limitation of this study was that we reported a relatively small series from a single tertiary institution limiting the statistical power and generalizability of this analysis. The retrospective nature of the study may have introduced errors in classifying adverse events. Moreover, the wide heterogeneity in radiotherapy courses delivered in the cohort posed a challenge in isolating potential factors of adverse events during analysis. Thus, the safety of RT with ICI should be investigated further in future larger studies with a more homogeneous RT timing, target site, and dose regimens. However, this study adds to the literature that if needed patients can safely receive radiation around the use of PD1/PDL1 inhibitors. In line with the prolonged half-life of ICI agents and the mechanism of action of immune modulation, which does not immediately stop with drug interruption, delays in radiation or ICI initiation to increase the timing interval akin to what is recommended with chemotherapy may not be necessary.

## 5. Conclusions

RT within 14 and 90 days of ICI administration was not associated with increased Grade ≥2 toxicities compared to RT outside these intervals, with the caveat of small cohort size. The findings suggested the safety of RT around ICI use, and routine delay of either therapy may not be necessary.

## Figures and Tables

**Table 1:** Baseline, disease, and treatment characteristics in patients with advanced non-small-cell lung cancer receiving both immune checkpoint inhibitor (ICI) and radiotherapy (N = 64).

		Interval 1			Interval 2		
	Total (N = 64)	Within (N = 20)	Outside (N = 44)	*p* *	Within (N = 40)	Outside (N = 24)	*p* *
Age (years)				0.0091			0.3177
Median	70	65	73		69	71	
Interquartiles	64–77	61–69	67–79		63–76	67–79	
Females (%)	29 (45%)	11 (55%)	18 (41%)	0.4171	21 (53%)	8 (33%)	0.1953
Histology (%)				0.6835			0.4598
Adenocarcinoma	56 (88%)	18 (90%)	38 (86%)		36 (90%)	20 (83%)	
Squamous cell carcinoma	8 (13%)	2 (10%)	6 (14%)		4 (10%)	4 (17%)	
Stage (AJCC 8th)				0.4968			0.4350
IVA-B	63 (98%)	20 (100%)	43 (98%)		39 (98%)	24 (100%)	
IIIB	1 (2%)	0 (0%)	1 (2%)		1 (2%)	0 (0%)	
ECOG performance score (%)				0.9684			0.6832
0	14 (22%)	5 (25%)	9 (20%)		10 (25%)	4 (17%)	
1	36 (56%)	11 (55%)	25 (57%)		20 (50%)	16 (67%)	
2	11 (17%)	3 (15%)	8 (18%)		8 (20%)	3 (12%)	
3	3 (5%)	1 (5%)	2 (5%)		2 (5%)	1 (4%)	
Ethnicity (%)				0.2150			0.0140
Caucasian	50 (78%)	13 (65%)	37 (84%)		27 (68%)	23 (96%)	
East Asian	8 (13%)	4 (20%)	4 (20%)		8 (20%)	0 (0%)	
Others	6 (9%)	3 (15%)	3 (15%)		5 (12%)	1 (4%)	
Smoking history (%)				0.8327			0.7158
Yes	48 (75%)	16 (80%)	32 (73%)		31 (78%)	17 (71%)	
No	15 (23%)	4 (20%)	11 (25%)		8 (20%)	7 (29%)	
Unknown	1 (2%)	0 (0%)	1 (2%)		1 (2%)	0 (0%)	
PD-L1 status (%)				0.2126			0.0301
<1%	9 (14%)	1 (5%)	8 (18%)		3 (7%)	6 (25%)	
1–49%	11 (17%)	6 (30%)	5 (12%)		8 (20%)	3 (13%)	
≥50%	32 (50%)	9 (45%)	23 (52%)		18 (45%)	14 (58%)	
Unknown	12 (19%)	4 (20%)	8 (18%)		11 (28%)	1 (4%)	
EGFR status (%)				0.9245			0.6472
Positive	9 (14%)	3 (15%)	6 (14%)		7 (17%)	2 (8%)	
Negative	41 (64%)	12 (60%)	29 (66%)		25 (63%)	16 (67%)	
Unknown	14 (22%)	5 (25%)	9 (25%)		8 (20%)	6 (25%)	
ALK status (%)				0.1005			0.3433
Positive	1 (2%)	0 (0%)	1 (2%)		0 (0%)	1 (4%)	
Negative	48 (75%)	12 (60%)	36 (82%)		29 (73%)	19 (79%)	
Unknown	15 (23%)	8 (40%)	7 (16%)		11 (27%)	4 (17%)	
Prior chemotherapy (%)				0.8713			0.2409
Yes	57 (89%)	18 (90%)	39 (89%)		34 (85%)	23 (96%)	
No	7 (11%)	2 (10%)	5 (11%)		6 (15%)	1 (4%)	
Prior targeted therapy (%)				0.1649			0.8247
Yes	6 (9%)	0 (0%)	6 (14%)		4 (10%)	2 (8%)	
No	58 (91%)	20 (100%)	38 (86%)		36 (90%)	22 (92%)	
ICI agent (%)				0.3632			0.0391
Nivolumab	29 (45%)	10 (50%)	19 (43%)		19 (48%)	10 (42%)	
Pembrolizumab	24 (38%)	9 (45%)	15 (34%)		18 (45%)	6 (25%)	
Atezolizumab	6 (9%)	0 (0%)	6 (14%)		1 (2%)	5 (21%)	
Durvalumab	5 (8%)	1 (5%)	4 (9%)		2 (5%)	3 (12%)	
Duration of ICI use (months)				0.4647			0.6703
Median	4.2	5.6	3.7		3.9	4.4	
Interquartiles	2.1–12.8	2.8–12.7	1.5–12.9		2.3–12.7	1.5–12.9	
Steroid use after ICI (%)	26 (41%)	3 (15%)	35 (80%)	<0.0001	16 (40%)	22 (92%)	<0.0001
Radiotherapy dose regimen (%)				0.7389			0.3819
Palliative	16 (25%)	5 (25%)	11 (25%)		11 (28%)	5 (21%)	
Radical conventional	10 (16%)	2 (10%)	8 (18%)		4 (10%)	6 (25%)	
Ablative	9 (14%)	4 (20%)	5 (11%)		7 (17%)	2 (8%)	
More than 1 regimen above	29 (45%)	9 (45%)	20 (45%)		18 (45%)	11 (46%)	
Radiotherapy technique				0.7490			0.2000
2/3D-CRT	8 (13%)	2 (10%)	6 (14%)		5 (13%)	3 (13%)	
VMAT/IMRT/GammaKnife	29 (45%)	8 (40%)	21 (48%)		15 (38%)	14 (58%)	
Both	27 (42%)	10 (50%)	17 (39%)		20 (50%)	7 (29%)	
Radiotherapy target site (%)				0.3904			0.2285
Intracranial only	4 (6%)	1 (5%)	3 (7%)		4 (10%)	0 (0%)	
Extracranial only	36 (56%)	9 (45%)	27 (61%)		20 (50%)	16 (67%)	
Both	24 (38%)	10 (50%)	14 (32%)		16 (40%)	8 (33%)	

* *p*-value was obtained by Wilcoxon rank-sum test for continuous variables or Fisher exact test for categorical variables. Two-sided value of *p* < 0.05 was considered statistically significant.

**Table 2:** All radiotherapy (RT) courses and crude Grade ≥2 toxicities among patients who received RT within Interval 1 (14 days) or Interval 2 (90 days) of immune checkpoint inhibitor use.

Variable	All Patients (N = 64)	Received RT in Interval 1 (N = 20)	No RT in Interval 1 (N = 44)	Received RT in Interval 2 (N = 40)	No RT in Interval 2 (N = 24)
Median RT courses prescribed (range)	3 (1–31)	5 (1–17)	2 (1–31)	5 (1–17)	2 (1–31)
Median RT prescription dose in Gy (range)	20 (8–78)	20 (8–78)	20 (8–66)	20 (8–78)	23 (8–66)
Median RT fractions per regimen (range)	2 (1–39)	2 (1–39)	2 (1–33)	2 (1–39)	4 (1–33)
Median BED10 in Gy10 (range)	50 (14–106)	50 (14–94)	50 (14–106)	53 (14–106)	50 (14–106)
Median BED10 within Intervals in Gy10 (range)	N/A	Interval 1: 35 (14–94)	N/A	Interval 2: 50 (14–106)	N/A
Patients with intracranial RT (%)	28 (44%)	11 (55%)	17 (39%)	20 (50%)	8 (33%)
Patients with Grade ≥2 toxicities (%)	18 (28%)	7 (35%)	11 (25%)	12 (30%)	6 (25%)

**Table 3:** Grade ≥2 toxicities among 64 patients with advanced NSCLC treated with both RT and ICI.

	Grade			
Toxicity	2	3	4	5
Pneumonitis	1 (2%)	5 (8%)	0 (0%)	0 (0%)
Nausea/emesis	2 (3%)	0 (0%)	0 (0%)	0 (0%)
Hepatitis	1 (2%)	1 (2%)	0 (0%)	0 (0%)
Dermatitis	1 (2%)	0 (0%)	0 (0%)	0 (0%)
Pericardial effusion	0 (0%)	1 (2%)	0 (0%)	0 (0%)
Thrombocytopenia	0 (0%)	0 (0%)	1 (2%)	0 (0%)
Hypothyroidism	0 (0%)	1 (2%)	0 (0%)	0 (0%)
Colitis	0 (0%)	1 (2%)	0 (0%)	0 (0%)
Encephalitis	0 (0%)	0 (0%)	0 (0%)	1 (2%)
Arthritis	0 (0%)	1 (2%)	0 (0%)	0 (0%)
Adrenitis	1 (2%)	0 (0%)	0 (0%)	0 (0%)
Esophagitis	1 (2%)	0 (0%)	0 (0%)	0 (0%)
Fever	0 (0%)	1 (2%)	0 (0%)	0 (0%)

**Table 4:** Univariable logistic regression analysis of potential predictors of Grade ≥2 toxicities.

Variable	OR	95% CI		*p*-Value
Age	1.01	0.96	1.08	0.54
Histology (SCC vs. Adenocarcinoma)	3.00	0.64	14.29	0.15
ECOG score				0.27
0 vs. ≥2	1.64	0.23	14.26	0.88
1 vs. ≥2	3.39	0.77	24.00	0.11
PD-L1 status				0.33
<1% vs. ≥50%	1.79	0.32	8.83	0.97
1–49% vs. ≥50%	2.98	0.68	13.12	0.27
RT within 14 days of ICI use (Interval 1)	1.62	0.50	5.07	0.41
RT within 90 days of ICI use (Interval 2)	1.29	0.42	4.26	0.67
RT regimen within Interval 1				0.21
Ablative vs. Palliative	1.28	0.18	8.98	0.80
Radical conventional vs. Palliative	6.42	0.33	124.20	0.22
More than 1 regimen vs. Palliative	0.07	0.01	0.84	0.04 *
RT regimen within Interval 2				0.05
Ablative vs. Palliative	0.90	0.22	3.64	0.88
Radical conventional vs. Palliative	1.98	0.40	9.92	0.41
More than 1 regimen vs. Palliative	0.17	0.04	0.73	0.02 *
Intracranial RT in Interval 1	0.20	0.02	1.38	0.98
Intracranial RT in Interval 2	0.28	0.05	1.21	0.97

Significant results are denoted by asterisks (*).

**Table 5:** Multivariable logistic regression analyses of Grade >2 toxicities on: A. RT within 14 days of ICI use and B. RT within 90 days of ICI use, after adjusting for potential predictive factors.

A	OR	95% CI		*p*-Value	B	OR	95% CI		*p*-Value
RT within 14 days of ICI use (Interval 1)	2.35	0.47	15.31	0.34	RT within 90 days of ICI use (Interval 2)	1.79	0.40	8.93	0.48
Age	1.00	0.91	1.09	0.92	Age	0.98	0.90	1.07	0.71
Histology (SCC vs. Adenocarcinoma)	3.74	0.60	27.83	0.21	Histology (SCC vs. Adenocarcinoma)	3.79	0.63	26.85	0.20
ECOG score				0.36	ECOG score				0.36
0 vs. ≥2	2.62	0.22	79.96	0.91	0 vs. ≥2	2.01	0.18	45.05	0.93
1 vs. ≥2	5.53	0.58	181.76	0.15	1 vs. ≥2	4.79	0.55	108.46	0.15
PD-L1 status				0.20	PD-L1 status				0.18
<1% vs. ≥50%	2.12	0.36	12.67	0.41	<1% vs. ≥50%	2.05	0.36	11.71	0.42
1–49% vs. ≥50%	2.01	0.58	7.01	0.27	1–49% vs. ≥50%	2.11	0.62	7.20	0.23
RT regimen				0.12	RT regimen				0.12
Ablative vs. Palliative	0.74	0.14	3.78	0.71	Ablative vs. Palliative	0.65	0.12	3.52	0.62
Radical conventional vs. Palliative	0.70	0.14	3.48	0.67	Radical conventional vs. Palliative	0.75	0.15	3.70	0.72
More than 1 regimen vs. Palliative	0.33	0.08	1.31	0.11	More than 1 regimen vs. Palliative	0.35	0.09	1.37	0.13
Intracranial RT in Interval 1	0.95	0.19	4.55	0.95	Intracranial RT in Interval 2	0.92	0.19	4.56	0.93

## Data Availability

Data available on request due to privacy or ethical restrictions.
